# A summary of the rapid services changes made in response to staff psychological needs and maintaining care to those with cancer in light of COVID‐19

**DOI:** 10.1002/pon.5449

**Published:** 2020-07-07

**Authors:** Cari Davies, Sophie Campbell, Jane Hutton, Andrew Morgan, Mary Gemma Cherry

**Affiliations:** ^1^ Department of Psychological Sciences, Institute of Psychology Health and Society, University of Liverpool Liverpool UK; ^2^ Cancer Psychology Service The Royal Liverpool University Hospital, Liverpool University Hospitals Foundation Trust Liverpool UK

**Keywords:** cancer, COVID‐19, healthcare staff, oncology, patient care

1

Dear Psycho‐Oncology Editor,

The Cancer Psychology Service within the Liverpool University Hospitals Trust in the UK has put additional measures in place to support staff with changes in patient care throughout the COVID‐19 pandemic.

Under normal circumstances, the National Institute for Health and Care Excellence^1^ recommend a 4‐tier model of increasing level of psychological skills that health care professionals who provide cancer care must possess, depending on their level of expertise. Our cancer clinical nurse specialists (CNSs) undertake Level 2 psychological training based on these guidelines to support their patients. Subsequently, CNSs are skilled in recognising signs of distress and risk, offering support through problem‐solving, listening and exploration skills. Clinical supervision from a clinical psychologist is offered to CNSs on a regular basis to build on this initial training and allow space to reflect on the use of psychological skills. Usually staff would have regular face‐to‐face contact with patients and families, whereby any issues could be identified and resolved. Unfortunately, individuals are being told that their treatment is halted temporarily, resulting in less frequent check‐ups and increased reliance on phone rather than face‐to‐face consultations. Patients are less likely to have access to their usual support from the CNSs, including information and help with problem‐solving. As there are fewer investigations into new cancers and limited surgeries and treatments taking place, CNSs are having more difficult conversations, and are facing the challenge of managing conflict between providing optimal cancer care and minimising risk from the virus. These concerns, along with staff shortages and deployment, in addition to a lack of resources, can result in challenging ways of working. These demands could make staff vulnerable to moral injury ‐ psychological distress that occurs from actions, or the lack of actions that go against an individual's moral code.^2^, ^3^

In response to the challenges faced by patients and staff, the Trust has developed staff support hubs which are open 24 hours a day and provide a calm, supportive safe space for those who would like time away from their usual work environment. The hubs are also staffed during certain hours by trained volunteer staff helpers, who offer psychological first aid, listen to concerns and provide resources for further support. Additionally, all staff members can access one of 14 in‐house psychologists to support their mental health and psychological well‐being by, for example, helping them to reflect on strategies to cope effectively in the current climate whilst maintaining a degree of resilience. To date, 58 individuals have requested the service. The Trust has also made numerous resources available via a designated COVID‐19 staff intranet page, including information and practical resources for supporting staff with self‐care and managing stress. These include national resources and more local ones, such as an evidence‐based online workshop^4^ for the prevention of post‐traumatic stress disorder, which cover awareness of post‐traumatic responses and coping skills.

For oncology staff, in addition to the above support, approaches have been tailored through collaboration between the lead cancer nurses and the cancer psychology service to meet the needs of CNSs and oncology staff. Level 2 supervision is still being offered to CNS via telephone and video calls to reflect on specific issues. Additionally, specialist support is being offered to the CNSs by a team of three clinical psychologists who are experienced in dealing with psycho‐oncology related issues. Conversations are offered 1:1 or in groups over phone or video call and aim to assist staff to manage the psychological impact of these new challenges. Despite being regularly advertised, there has been no uptake of this service to date, feedback from CNSs suggests that it may be of more relevance once services resume because they anticipate that patients may experience elevated distress at this point.

Through collaboration with lead cancer nurses and Level 2 supervision, some common concerns have been identified. Consequently, a weekly bulletin was created to help CNSs to easily access resources and helpful strategies. The content of these bulletins varies from general wellbeing support and techniques to more specific content dependent on themes arising during staff discussions. The first bulletin referred to personal wellbeing plans, with a focus on using mindfulness to cultivate awareness of thoughts and feelings and to help notice possible signs of stress and anxiety. A guide was included on how to manage difficult conversations and the related emotional demands. The second bulletin discussed the demand for CNSs to adjust to new ways of working with reflections made on the adjustment process and the way this may manifest. Common worries raised in supervision sessions—including concerns around personal health, family and new ways of working ‐ were shared via the bulletin to help normalise and validate responses to these challenges. The third bulletin focused on ways of dealing with emotional and physical fatigue, as well as ways to improve sleep hygiene. The fourth bulletin aimed to encourage maximising peer support, and the value of keeping in contact with loved ones when usual support mechanisms are not readily available. The fifth bulletin highlighted the overlap between stress and anxiety response with COVID‐19 symptoms, with encouragement to check in with the body to notice these. The sixth bulletin covered the importance of self‐care and self‐compassion in managing stress.

Regular emails have been sent by the service and Cancer Lead Nurse to encourage feedback and suggestions as to what CNS may find helpful to include in future bulletins. Initial feedback suggests that the approaches discussed have been well‐received and that staff needs are being recognised. CNSs valued the accessibility of Level 2 supervision and bulletins during the initial acute phase of the pandemic, but we anticipate that uptake of 1:1 support will increase as clinical services resume. The resources discussed are not static, and will be continually developed and evaluated to continue to meet the needs of staff. It is crucial that staffs are adequately supported with these increasing demands to ensure that those affected by cancer receive the best possible care throughout COVID‐19.

**Key Points**

1. Healthcare staff, patients and their families are facing many disruptions because of COVID‐19.

2. Liverpool University Hospitals NHS Foundation Trust is offering psychological support and well‐being hubs for all staff.

3. Cancer clinical nurse specialists are offered psychological support and continued Level 2 supervision by the cancer psychology service.

4. A weekly bulletin has been created to share psychological well‐being resources for oncology staff.

5. The available resources will be monitored and revised to meet the needs of staff and help maintain care for those affected by cancer.

2

## Data Availability

Data sharing is not applicable to this article as no new data were created or analyzed in this study.
